# Prevalence of and risk factors for Human Immunodeficiency Virus (HIV) infection in entrants and residents of an Ethiopian prison

**DOI:** 10.1371/journal.pone.0271666

**Published:** 2023-02-09

**Authors:** Eliyas Tsegaye Sahle, Wondwossen Amogne, Tsegahun Manyazewal, Jill Blumenthal, Sonia Jain, Shelly Sun, Jason Young, Eric Ellorin, Habtamu Woldeamanuel, Lemma Teferra, Beniam Feleke, Olivier Vandenberg, Zilma Rey, Melissa Briggs-Hagen, Richard Haubrich, John Allen McCutchan

**Affiliations:** 1 ADDIS-VP Project/Ethiopian Public Health Association, Addis Ababa, Ethiopia; 2 Université Libre de Bruxelles/Ecole de Sante Public, Brussels, Belgium; 3 College of Health Sciences, Addis Ababa University, Addis Ababa, Ethiopia; 4 University of California San Diego, San Diego, California, United States of America; 5 Ethiopian Federal Prison Administration, Addis Ababa, Ethiopia; 6 Centers for Disease Control and Prevention, Addis Ababa, Ethiopia; 7 Environmental and Occupational Health Research Centre (CRSET), School of Public Health, Université Libre de Bruxelles, Brussels, Belgium; 8 Division of Infection & Immunity, University College London, London, United Kingdom; 9 Centers for Disease Control and Prevention, Atlanta, GA, United States of America; 10 Gilead Sciences, Foster City, California, United States of America; University of North Carolina at Chapel Hill, UNITED STATES

## Abstract

**Background:**

Prisoners generally have a higher prevalence of HIV infection compared to the general population from which they come. Whether this higher prevalence reflects a higher HIV prevalence in those entering prisons or intramural transmission of HIV within prisons or both is unclear. Any of these possibilities would increase the prevalence found in resident prisoners above that in the general population. Moreover, comparisons of HIV prevalence in entrants and residents and in men and women in African prisons are not well documented. The purpose of this study was to estimate and compare the prevalence and risk factors for HIV infection amongst both male as well as female and entrant and resident prisoners in a large Ethiopian Federal Prison.

**Methods:**

We studied consenting prisoners cross-sectionally from August 2014 through November 2016. Prison entrants were screened continuously for HIV infection and its associated risk factors and residents were screened in two waves one year apart. HIV was diagnosed at the prison hospital laboratory based on the Ethiopian national HIV rapid antibody testing protocol. An external, internationally-accredited reference laboratory confirmed results. Agreement of results between the laboratories were assessed.

**Results:**

A total of 10,778 participants were screened for HIV. Most participants were young (median age of 26 years, IQR: 21–33), male (84%), single (61%), literate (89%), and urban residents (91%) without prior incarceration (96%). Prevalence of HIV was 3.4% overall. Rates of HIV (p = 0.80) were similar in residents and entrants in wave 1 and in entrants in both waves, but were 1.9-fold higher (5.4% vs 2.8%) in residents than entrants in wave 2 (both p<0.001). At entrance to the prison women were more likely to be HIV+ than men (5.5% in women vs 2.5% in men, p< 0.001). In contrast resident women were less likely to be HIV+, but this difference was not statistically significant (3.2% in women vs 4.3% in men, p = 0.125). Other risk factors associated with HIV infection were increasing age (p<0.001), female gender (p<0.001), marital status (never vs other categories, p = 0.016), smaller number of rooms in their houses pre-imprisonment (p = 0.031), TB diagnosis ever (p<0.001), number of lifetime sex partners (especially having 2–10, p<0.001), and genital ulcer (p = 0.037).

**Conclusions:**

Prevalence of HIV in the residents at this large, central Ethiopian prison was higher than that estimated for the general population and lower than in many other studies from other smaller Ethiopian prisons. A higher prevalence in residents than in entrants were found only in our second wave of screening after one year of continuous screening and treatment, possibly representing increased willingness of residents at increased risk of HIV to participate in the second wave. Thus, this findings did not clearly support intramural transmission of HIV or the effectiveness of screening to reduce prevalence. Finally, the higher HIV prevalence in women than men requires that they be similarly screened and treated for HIV infection.

## Introduction

HIV remains a major public health problem in spite of remarkable progress in diagnosis, prevention, and treatment of HIV infection over four decades [1]. Globally in 2020, approximately 37.6 million persons were living with HIV (PLHIV) and 1.5 million people were newly infected. The sub-Saharan region of Africa is the most affected with 20.6 million PLHIV in 2020 and 44.7% of the global total of new HIV infections.

Ethiopia is the second largest country in Africa with an estimated population of 111 million in 2021. It had a prison population of about 110,000 and an incarceration rate of about of 99 prisoners /10^5^ population and about 4.2% of them are women [2].

HIV is endemic in Ethiopia with declining prevalence rates for both from 2005 to 2014 [3]. The 2014 HIV Sentinel Surveillance Study of women in antenatal care, a sample of the general population that minimizes some selection biases, found an overall HIV prevalence of 2.0% with less in rural areas (1.4%) than in cities (3.9%). The estimated number of HIV-infected persons was 928,000 or less than 1% of the population.

HIV is transmitted primarily sexually in most settings, may be asymptomatic, and provokes long-lived antibody responses that can identify latent (asymptomatic) infection. Prisoners have higher rates of HIV than the populations in their catchment [4]. For these reasons prisons are targeted for HIV screening in many countries [4, 5]. Intramural transmission may contribute to this increased prevalence in prisons. Intramural transmission of HIV in prisons by sex, injection drug use, tattooing, or nosocomial routes may contribute to this increased prevalence. Ethiopian prison populations have had a higher prevalence of HIV than the general population throughout the HIV epidemic. For example, in the Dire Dawa District prison in 1991, HIV antibody prevalence was 6.0% compared to 3.3% in urban populations [6].

In an unpublished pilot study at Kality Prison in 2011, we found that HIV prevalence was 4-fold higher in resident prisoners (42/277 = 15%) than in entrants (10/279 = 3.6%) supporting intramural transmission of HIV. Based on this finding, we designed this study to provide more precise estimates of HIV prevalence at Kality. Our aims were to compare the prevalence of HIV at Kality to rates in: a) the general Ethiopian population, b) between entrant and resident prisoners, and c) between men and women at Kality Prison, and d) in residents before and after a period of enhanced screening of both entrants and residents. We also wanted to identify risk factors for HIV in these prisoners.

Thus, we performed cross-sectional serologic screening for HIV in a continuous sample of entrants and in prison residents in two waves one year apart.

These goals were part of a larger study screening for the prevalence of another common infection in prisoners, active pulmonary tuberculosis (TB) [7].

We hypothesized that residents would have a higher prevalence than entrants which would support intramural transmission of HIV. A second hypotheses was that enhanced HIV screening and treatment of the prisoners might reduce its transmission and prevalence over the course of the study.

## Methods

### Study setting

This study was embedded in an observational study of the prevalence of tuberculosis, HIV and syphilis sposnsored by the US Centers of Disease control. We studied consenting prisoners in Kality Prison, a high-security federal prison, located 15 kilometers from central Addis Ababa, the capital of Ethiopia. It provides long-term incarceration, shorter-term detention before and during trials, and temporary holding of prisoners before their transfer to other federal prisons. It consists of: a) a central high-security area divided into eight zones, b) an adjacent lower security area containing medical clinics that manage HIV, syphilis, and TB, c) two inpatient wards with a total of 58 beds, d) a 40-bed TB and general medical ward, and e) a well-equipped clinical laboratory and digital x-ray facility. Study personnel had access only to the latter areas.

During our study an average of about 60 new prisoners entered the prison daily and the resident prisoner census averaged about 4,500. However, we had no access to more detailed census data. Full-time medical staff at Kality consists of 3 physicians, 9 health officers (physician’s assistants), 40 nurses, 6 laboratory technicians, 3 radiography technicians who both obtained and read radiographs and a part-time consulting radiologist. Clinics specific for TB, HIV and other STIs, manage all prisoners with these diagnoses according to Ethiopian national guidelines.

### Recruitment and enrollment

Potential participants were recruited by prison medical staff either during health screening at entry (entrants) or at *ad hoc* group meetings within each prison zone (residents). Full-time, prison health care workers who were trained in ethical conduct of research obtained written informed consent by providing information to groups of 5–30 prisoners followed by individual interviews to answer additional questions. Prisoners were informed that they could accept, refuse or discontinue participating in the study, without affecting their health care, imprisonment or parole.

### Study design

We initiated two independent, cross-sectional waves of screening of residents (R) and continuous, but not universal, screening of newly-admitted, entrant (E) prisoners for TB and HIV from August 2014 through November 2016. We were unable to recruit or interview entrants who arrived when research staff were unavailable. Residents, excluding those who had been examined as entrants, were screened in two waves: (R1 = 8/2014 to 4/2015) and (R2 = 11/2015 to 11/2016), Entrants screened during these waves were designated E1 and E2 and those screened in the gap between waves were designated entrants screened in the gap period (EG).

### Data collection

Demographic data including information on risk factors for HIV and syphilis were collected using a structured questionnaire developed in English, translated to the Ethiopian national language (Amharic) and validated through back-translation into English. Data was collected by trained health care workers and recorded on paper case report forms (CRFs) with a unique participant identification number.

Data collected using the paper CRFs was directly entered into the University of California, San Diego (UCSD)-based database OCCAMS (Open-source Clinical Content Analysis and Management System) by trained data entry clerks at Kality and verified by a full-time data manager. Data quality was completely audited (100%) by the study data manager. The investigators reviewed regular tracking reports of internally accumulating data generated by the UCSD-based statistical team. The data was also assessed by the Centers for Disease Control and Prevention (CDC) three times and by an independent auditor twice during the study. Formal reporting of audits identified deficiencies and errors to which the study team responded with appropriate corrections and/or methodological adjustments.

Variables collected as potential risk factors for HIV before or after imprisonment were:

*Socio-demographic*. Age, gender, self-identified ethnicity, education, literacy, marital status, occupation, and religion.

*Medical*. TB diagnosis ever, diabetes diagnosis ever, cancer diagnosis ever, malnutrition, urinary pain (dysuria), scrotal swelling, genital discharge, genital ulcer, syphilis infection (at Kality and ICL labs), HIV infection (at Kality lab),

*Behavioral*. Current smoking, alcohol use prior prison, current use of Khat, and number of lifetime sex partners.

*Prison-related conditions*. Residence prior to prison (urban/rural), prison location (entrant/resident), prior imprisonment, prior prison duration, and current prison duration.

*Housing conditions before prison*. Number of people in the house and number of rooms in the house.

### Serological testing

All subjects who consented for HIV had a rapid HIV test performed at the Kality laboratory. HIV rapid testing followed the standard prison algorithm of sequential testing following the national guidelines [8]. I Initial testing used KHB (Shanghai Kehua Bioengineering, Ltd, Shanghai, China) kit [9], subsequent confirmation with HIV1/2 STAT-PAK (CHEMBIO Diagnostic systems, Inc., Medford, NY, USA) [10] and resolution of discordance with Uni-Gold (Trinity Biotech, Jamestown, NY, USA). All tests used an immunochromatographic assay method with various levels of sensitivity and specificity [11] (Table 1).

**Table 1:** Published sensitivity and specificity of standard test kits used at Kality Prison Laboatory with performance references from package inserts.

HIV Test kit [Reference]	Sensitivity	Specificity
• **KHB [14]**	100%	100%
• **STAT-PAK [15]**	99.7%	99.3%
• **Uni-Gold [16]**	100%	100%

### Clinical care and treatment

HIV positive cases were referred and linked to the ART clinic in Kality for treatment, care and follow-up per the national guidelines [12].

### Ethical and regulatory considerations

This study was designed to assess screening procedures that directly benefit prisoners by diagnosing treatable diseases common in Ethiopian prisons. It was conducted after protocol review by the US Centers for Disease Control and the Ethiopian Federal Prison Administration and by the Institutional Review Boards of the National Ministry of Science and Technology of Ethiopia, the Ethiopian Public Health Association and the University of California, San Diego. Informed written consent was obtained from all study participants. Those who were unwilling to undergo some of the procedures (eg, blood drawing or HIV testing) were included, but excused from those procedures.

### Data analysis

Data entered into OCCAMS were exported to the SPSS version 20.0 software program for analysis. Descriptive summaries were provided by study groups (E1, E2, EG, R1 and R2) and overall. Prevalence rates of HIV were compared between entrants and residents (E1 vs R1, E2 vs R2) using Chi-square and Fisher’s exact test. Sensitivity and specificity were calculated to assess accuracy or diagnostic performance. Multivariable logistic regression models were conducted to study the risk factors associated with HIV infection.

## Results

### Socio-demographic characteristics of participants

Of a total 13,803 prisoners enrolled in a study of screening for tuberculosis and HIV, 10,714 (78%) consented to HIV testing. Participants were 83.9% men and 16.1% women, young (median age: 26 years, IQR: 21–33), single (61%), literate (89%), and urban residents (91%) without prior incarceration (96%) (Table 2).

**Table 2:** Associations of HIV prevalence with socio-demographic and medical characteristics and history of incarceration and sexual behavior for all the participants and in entrants and residents separately.

Participant Characteristics	Tested for HIV total (N = 10,714) n (%)	HIV prevalence for each category n (% for all) and p values	HIV prevalence in Entrants n (%)	HIV prevalence in Residents n (%)	Prevalence differences in men and women p-value
**Prisoner category**		***P<0*.*001***			
Entrant	6019 (56.2)	170 (2.8)	-	-	-
Resident	4695 (43.8)	190 (4.0)	-	-	-
**Gender**		***p = 0*.*046***			-
Male	8986 (83.9)	288 (3.2)	130/5297 (2.5)	158/3689 (4.3)	**p<0.001**
Female	1728 (16.1)	72 (4.2)	40/722 (5.5)	32/1006 (3.2)	**p = 0.02**
**Age** *(Median*, *IQR)*	*26 (21–33)*	*-*	*25 (21–31)*	*27 (21–35)*	
**Age Categories**		***p<0*.*001***			
18–20	2548 (23.8)	30 (1.2)	16/1501(1.1)	14/1047 (1.3)	p = 0.578
21–25	2760 (25.8)	49 (1.8)	26/1700 (1.5)	23/1060 (2.2)	p = 0.237
26–30	2204 (20.6)	69 (3.1)	32/1268 (2.5)	37/936 (4.0)	p = 0.063
31–35	1095 (10.2)	57 (5.2)	28/603 (4.6)	29/492 (5.9)	p = 0.412
36–40	752 (7.0)	53 (7.0)	22/390 (5.6)	31/362 (8.6)	p = 0.153
41–45	416 (3.9)	40 (9.6)	17/188 (9.0)	23/228 (10.1)	p = 0.742
≥ 46	939 (8.8)	62 (6.6)	29/369 (7.9)	33/570 (5.8)	p = 0.227
**Ethnicity**		***p = 0*.*009***			
Amhara	4111 (38.4)	157 (3.8)	67/2280 (2.9)	90/1831 (4.9)	**p = 0.001**
Oromo	2575 (24.0)	94 (3.7)	44/1434 (3.1)	50/1141 (4.4)	p = 0.090
Tigre	1038 (9.7)	39 (3.8)	18/534 (3.4)	21/504 (4.2)	p = 0.518
Southern	2204 (20.6)	52 (2.4)	36/1516 (2.4)	16/688 (2.3)	p = 1.000
Others	786 (7.3)	18 (2.3)	5/255 (2.0)	13/531 (2.4)	p = 0.802
**Education**		***p = 0*.*009***			
None	1216 (11.3)	43 (3.5)	27/770 (3.5)	16/446 (3.6)	p = 1.000
Elementary (1–8)	4689 (43.8)	146 (3.1)	68/2621 (2.6)	78/2068 (3.8)	**p = 0.022**
Secondary (9–10)	2508 (23.4)	73 (2.9)	34/1431 (2.4)	39/1077 (3.6)	p = 0.072
Preparatory (11–12)	1090 (10.2)	51 (4.7)	26/594 (4.4)	25/496 (5.0)	p = 0.666
University (Diploma)	709 (6.6)	36 (5.1)	11/366 (3.0)	25/343 (7.3)	**p = 0.010**
University (Undergrad)	429 (4.0)	9 (2.1)	3/210 (1.4)	6/219 (2.7)	p = 0.504
Post-grad (MS/PhD)	73 (0.7)	2 (2.7)	1/27 (3.7)	1/46 (2.2)	p = 1.000
**Occupation**		***p = 0*.*001***			
None	315 (2.9)	9 (2.9)	4/170 (2.4)	5/145 (3.4)	p = 0.737
Student	1161 (10.8)	20 (1.7)	8/563 (1.4)	12/598 (2.0)	p = 0.504
Housewife	116 (1.1)	8 (6.9)	7/61 (11.5)	1/55 (1.8)	p = 0.064
Merchant	1101 (10.3)	53 (4.8)	23/517 (4.4)	30/584 (5.1)	p = 0.673
Gov. employee	814 (7.6)	26 (3.2)	8/402 (2.0)	18/412 (4.4)	p = 0.071
Peasant	614 (5.7)	17 (2.8)	8/306 (2.6)	9/308 (2.9)	p = 1.000
Other	6593 (61.5)	227 (3.4)	112/4000 (2.8)	115/2593 (4.4)	**p<0.001**
**Marital Status**		***p<0*.*001***			
Never Married	6541 (61.1)	144 (2.2)	73/3878 (1.9)	71/2663 (2.7)	**p = 0.039**
Married	3861 (36.0)	180 (4.7)	85/2016 (4.2)	95/1845 (5.1)	p = 0.194
Divorced/Separated	199 (1.9)	28 (14.1)	9/88 (10.2)	19/111 (17.1)	p = 0.218
Widow/Widower	49 (0.5)	5 (10.2)	2/25 (8.0)	3/24 (12.5)	p = 0.667
Other	64 (0.6)	3 (4.7)	1/12 (8.3)	2/52 (3.8)	p = 0.470
**Residence prior to prison**		***p = 0*.*206***			
Urban	9739 (90.9)	318 (3.3)	167/5720 (2.9)	151/4019 (3.8)	**p = 0.024**
Rural	971 (9.1)	42 (4.3)	3/298 (1.0)	39/673 (5.8)	**p<0.001**
Pastoral	3 (0.0)	0 (0)	-	-	
^**†**^ **Number of rooms in house**		**p = 0.117**			
1	5247 (50.0)	190 (3.6)	116/3604 (3.2)	74/1643 (4.5)	**p = 0.025**
2–5	4554 (42.5)	141 (3.1)	46/2056 (2.2)	95/2498 (3.8)	**p = 0.003**
6–10	832 (7.5)	29 (3.5)	8/337 (2.4)	21/495 (4.2)	p = 0.179
>10	-	-	-	-	-
**Prisoner category**		***p = 0*.*002***			
Entrant	6019 (56.2)	170 (2.8)	-	-	-
Resident	4695 (43.8)	190 (4.0)	-	-	-
**Prior Imprisonment**		***p = 0*.*088***			
No	10312 (96.2)	340 (3.3)	164/5866 (2.8)	176/4446 (4.0)	**p = 0.001**
Yes	402 (3.8)	20 (5.0)	6/153 (3.9)	14/249 (5.6)	p = 0.490
**Current Prison Duration**		***p = 0*.*011***			
≤ 5 years	9889 (92.5)	321 (3.2)	170/6011 (2.8)	151/3878 (3.9)	**p = 0.004**
5.1–10 years	659 (6.2)	29 (4.4)	0/8 (0.0)	29/651 (4.5)	p = 1.000
10.1–15 years	106 (1.0)	9 (8.5)	-	9/106 (8.5)	-
≥ 15.1 years	34 (0.3)	1 (2.9)	-	1/34 (2.9)	-
**Number Lifetime Sex Partners**	n = 9,227	***p<0*.*001***			
No partners	1267 (13.7%)	20 (1.6)	9/727 (1.2)	11/540 (2.0)	p = 0.265
1	4071 (44.1%)	114 (2.8)	70/2656 (2.6)	44/1415 (3.1)	p = 0.425
2–10	3387 (36.7%)	153 (4.5)	74/1805 (4.1)	79/1582 (5.0)	p = 0.215
11 and above	502 (5.4%)	25 (5.0)	4/136 (16.0)	21/366 (5.7)	p = 0.252
**Genital discharge**		***p = 0*.*002***			
No	10236 (95.5)	331 (3.2)	165/5938 (2.8)	166/4298 (3.9)	p = 0.003
Yes	478 (4.5)	29 (6.1)	5/81 (6.2)	24/397 (6.0)	p = 1.000
**Genital ulcer**		***p<0*.*001***			
No	10413 (97.2)	337 (3.2)	166/5958 (2.8)	171/4455 (3.8)	p = 0.003
Yes	301 (2.8)	23 (7.6)	4/61 (6.6)	19/240 (7.9)	p = 1.000
**Scrotal swelling**		***p = 0*.*012***			
No	10554 (98.5)	348 (3.3)	169/5996 (2.8)	179/4558 (3.9)	**p = 0.002**
Yes	160 (1.5)	12 (7.5)	1/23 (4.3)	11/137 (8.0)	p = 1.000
**Dysuria (Urinary pain)**		***p = 0*.*086***			
No	9509 (88.8)	309 (3.2)	161/5792 (2.8)	148/3717 (4.0)	**p = 0.002**
Yes	1205 (11.2)	51 (4.2)	9/227 (4.0)	42/978 (4.3)	p = 1.000

### Sero-prevalence of and risk factors for HIV infection in all participants and separately in entrants and residents

The prevalence of HIV based on the rapid testing algorithm at Kality lab in all participants was 3.4% (360/10,714) (Table 2). We compared all 360 HIV-positive (HIV+) to the 10,354 HIV-negative (HIV-) prisoners for risk factors in bivariate analysis (Table 2). HIV prevalence increased as the number of lifetime sexual partners increased from 0 to ≥11 partners (1.6% to 5.0%, p<0.001) and risk was especially high in those having 2–10 sex partners (AOR = 1.6, p<0.001). Prevalence of HIV was also significantly higher among participants reporting symptoms of other STIs: genital ulcer (7.6% vs 3.2%, p<0.001), genital discharge (6.1% vs 3.2%, p = 0.002), and scrotal swelling (6.9% vs 3.1%, p = 0.012. (Table 2). Contrary to expectations based on dysuria being a symptom of gonococcal or chlamydial urethritis, it was associated with reduced risk of HIV infection (p = 0.007).

HIV prevalence in all entrants was 2.8% (170/6019), and in all residents was 4.0% (190/4695), a 1.4 fold difference (p<0.001). We compared differences in percentage to To see if patient characteristics HIV prevalence in entrants (E) and residents (R) differed from those in all participants we compared their rates by sociodemographic categories (Table 2). Prevalence of HIV was greater in resident than entrant men (E = 2.5% < R = 4.3, p < 0.001), but in women HIV prevalence was greater in entrants than residents (E = 5.5 > R = 3.2, p = 0.02). Differences in HIV prevalence were found in several ethnic groups, but was significant only in the largest group, the Amhara (E = 2.9 < R = 4.9, p = 0.001).

HIV prevalence differed in only two educational strata: the largest group with 1–8 years of education (E = 2.6 < R = 2.8, p = 0.022) and those with a university diploma (E = 3.0 < R = 7.3, p = 0.010). Of major occupational categories, only the largest category (“other,” not further defined) had a significantly increased prevalence in residents (R = 4.4 > E = 2.8, p = 0.001. Two other occupational groups differed with borderline significance: housewives (E = 11.5 > R = 1.8, p = 0.064) and government employees (E = 2.0 < R = 4.4, p = 0.071) and in all occupational categories residents had more prevalence HIV than entrants. Of groups classified ed by marital status, only the largest group, “never married”, differed significantly (E = 1.9 < R = 2.7, p = .039).

Resident prisoners from both urban and rural places of residence before prison had higher HIV prevalence that entrants: urban E = 2.9 < R = 3.8, p = 0.024, and rural E = 1.0 < R = 5.8, p <0.001. Prisoners who came from homes with either 1 or 2–5 rooms, had the expected higher rates in residents and then entrants: 1 room (E = 3.2 < R = 4.5, p = 0.025) and 2–5 rooms (E = 2.2 < R = 3.8, p = 0.03). Other characteristics with an expected, statistically significantly greater HIV prevalence in residents than entrants included history of prior imprisonment and current number of years in prison.

The prevalence of HIV during and in the gap (= G) between each of the two waves screening (1 or 2) by prisoner status (entrant = E or resident = R) were 2.2% in E1, 2.8% in E2, 2.7% in EG, 2.4% in R1, and 5.4% in R2 (Table 3). In the first wave, HIV prevalence was similar in residents (R1) and entrants (E1) (2.4% vs 2.2%, p = 0.80), but in the second wave was higher in residents (R2) than entrants (E2) (5.4% vs 2.7%, p<0.001) (Table 3). Thus, HIV prevalence was borderline higher in E2 than in E1 (2.7%/2.2% or 1.3-fold, p = 0.07),but strikingly higher (2.3 fold) in residents higher in R2 than R1 (5.4%/2.4% p<0.001).

**Table 3:** Associations of HIV prevalence with socio-demographic and medical characteristics and history of incarceration and sexual behavior for all the participants and separately in men and women.

Participant Characteristics	HIV tested (Total N = 10,714) n (%)	Overall HIV prevalence n (% for all) and p value for each category	HIV prevalence in men n (%)	HIV prevalence in women n (%)	Prevalence difference between men and women (p-value)
**Prisoner category**		***P<0*.*001***			
Entrant	6019 (56.2)	170 (2.8)	130/5297 (2.5)	40/722 (5.5)	**p<0.001**
Resident	4695 (43.8)	190 (4.0)	158/3689 (4.3)	32/1006 (3.2)	p = 0.125
**Gender**		***p = 0*.*046***			-
Male	8986 (83.9)	288 (3.2)	na	na	na
Female	1728 (16.1)	72 (4.2)	-na	na	na
**Age** *(median years*, *IQR)*	*26 (21–33)*	*-* ***p<0*.*001***	*27 (22–34)*	*21 (19–28)*	p = 0.046
18–20	2548 (23.8)	30 (1.2)	16/1726 (0.9)	14/822 (1.7)	p = 0.114
21–25	2760 (25.8)	49 (1.8)	37/2379 (1.6)	12/381 (3.1)	**p = 0.036**
26–30	2204 (20.6)	69 (3.1)	58/2002 (2.9)	11/202 (5.4)	p = 0.056
31–35	1095 (10.2)	57 (5.2)	43/992 (4.3)	14/103 (13.6)	**p<0.001**
36–40	752 (7.0)	53 (7.0)	41/670 (6.1)	12/82 (14.6)	**p = 0.010**
41–45	416 (3.9)	40 (9.6)	36/364 (9.9)	4/52 (7.7)	p = 0.803
≥ 46	939 (8.8)	62 (6.6)	57/853 (6.7)	5/86 (5.8)	p = 1.000
**Ethnicity**		***p = 0*.*009***			
Amhara	4111 (38.4)	157 (3.8)	127/3269 (3.9)	30/842 (3.6)	p = 0.762
Oromo	2575 (24.0)	94 (3.7)	72/2181 (3.3)	22/394 (5.6)	**p = 0.039**
Tigre	1038 (9.7)	39 (3.8)	34/926 (3.7)	5/112 (4.5)	p = 0.602
Southern	2204 (20.6)	52 (2.4)	43/1936 (2.2)	9/268 (3.4)	p = 0.279
Others	786 (7.3)	18 (2.3)	12/674 (1.8)	6/112 (5.4)	**p = 0.032**
**Education**		***p = 0*.*009***			
None	1216 (11.3)	43 (3.5)	30/922 (3.3)	13/293 (4.4)	p = 0.365
Elementary (0–8)	4689 (43.8)	146 (3.1)	120/3882 (3.1)	26/807 (3.2)	p = 0.824
Secondary (9–10)	2508 (23.4)	73 (2.9)	62/2204 (2.8)	11/304 (3.6)	p = 0.464
Preparatory (11–12)	1090 (10.2)	51 (4.7)	42/967 (4.3)	9/123 (7.3)	p = 0.169
University (Diploma)	709 (6.6)	36 (5.1)	27/604 (4.5)	9/105 (8.6)	p = 0.090
University (Undergrad)	429 (4.0)	9 (2.1)	6/348 (1.7)	3/81 (3.7)	p = 0.380
Post-grad (MS/PhD)	73 (0.7)	2 (2.7)	1/59 (1.7)	1/14 (7.1)	p = 0.349
**Occupation**		***p = 0*.*001***			
None	315 (2.9)	9 (2.9)	3/251 (1.2)	6/64 (9.4)	**p = 0.003**
Student	1161 (10.8)	20 (1.7)	17/983 (1.7)	3/178 (1.7)	p = 1.000
Housewife	116 (1.1)	8 (6.9)	1/5 (20.0)	7/111 (6.3)	p = 0.305
Merchant	1101 (10.3)	53 (4.8)	42/966 (4.3)	11/135 (8.1)	p = 0.081
Gov. employee	814 (7.6)	26 (3.2)	21/705 (3.0)	5/109 (4.6)	p = 0.377
Peasant	614 (5.7)	17 (2.8)	16/539 (3.0)	1/75 (1.3)	p = 0.709
Other	6593 (61.5)	227 (3.4)	188/5537 (3.4)	39/1056 (3.7)	p = 0.645
**Marital Status**		***p<0*.*001***			
Never Married	6541 (61.1)	144 (2.2)	113/5522 (2.0)	31/1019 (3.0)	p = 0.062
Married	3861 (36.0)	180 (4.7)	152/3246 (4.7)	28/615 (4.6)	p = 1.000
Divorced/Separated	199 (1.9)	28 (14.1)	16/139 (11.5)	12/60 (20.0)	p = 0.124
Widow/Widower	49 (0.5)	5 (10.2)	4/26 (15.4)	1/23 (4.3)	p = 0.353
Other	64 (0.6)	3 (4.7)	3/53 (5.7)	0/11 (0)	p = 1.000
**Residence prior to prison**		***p = 0*.*206***			
Urban	9739 (90.9)	318 (3.3)	250/8173 (3.1)	68/1566 (4.3)	**p = 0.013**
Rural	971 (9.1)	42 (4.3)	38/809 (4.7)	4/162 (2.5)	p = 0.288
Pastoral	3 (0.0)	0 (0)	0/3 (0)	0 (0)	-
**Rooms in house (n)**		**p = 0.117**			
1	5247 (50.0)	190 (3.6)	150/4579 (3.3)	40/668 (6.0)	**p = 0.001**
2–5	4554 (42.5)	141 (3.1)	120/3693 (3.2)	21/861 (2.4)	p = 0.231
6–10	832 (7.5)	29 (3.5)	18/652 (2.8)	11/180 (6.1)	**p = 0.038**
**Prisoner category**		***p = 0*.*002***			
Entrant	6019 (56.2)	170 (2.8)	130/5297 (2.5)	40/722 (5.5)	**p<0.001**
Resident	4695 (43.8)	190 (4.0)	158/3689 (4.3)	32/1006 (3.2)	p = 0.125
**Prior Imprisonment**		***p = 0*.*088***			
No	10312 (96.2)	340 (3.3)	272/8637 (3.1)	68/1675 (4.1)	p = 0.059
Yes	402 (3.8)	20 (5.0)	16/349 (4.6)	4/53 (7.5)	p = 0.318
**Current Prison Duration (years)**		***p = 0*.*011***			
≤ 5	9889 (92.5)	321 (3.2)	253/8269 (3.1)	68/1620 (4.2)	**p = 0.021**
5.1–10	659 (6.2)	29 (4.4)	26/561 (4.6)	3/98 (3.1)	p = 0.603
10.1–15	106 (1.0)	9 (8.5)	8/101 (7.9)	1/5 (20.0)	p = 0.364
≥ 15.1	34 (0.3)	1 (2.9)	1/32 (3.1)	0/2 (0)	p = 1.000
**Lifetime Sex Partners**	n = 9,227	***p<0*.*001***			
No partners	1267 (13.7%)	20 (1.6)	16/958 (1.7)	4/309 (1.3)	p = 0.796
1	4071 (44.1%)	114 (2.8)	78/3097 (2.5)	36/974 (3.7)	p = 0.058
2–10	3387 (36.7%)	153 (4.5)	131/3154 (4.2)	22/233 (9.4)	**p<0.001**
11 and above	502 (5.4%)	25 (5.0)	22/487 (4.5)	3/15 (20.0)	**p = 0.033**
**Genital discharge**		***p = 0*.*002***			
No	10236 (95.5)	331 (3.2)	264/8594 (3.1)	67/1642 (4.1)	**p = 0.037**
Yes	478 (4.5)	29 (6.1)	24/392 (6.1)	5/86 (5.8)	p = 1.000
**Genital ulcer**		***p<0*.*001***			
No	10413 (97.2)	337 (3.2)	266/8710 (3.1)	71/1703 (4.2)	**p = 0.018**
Yes	301 (2.8)	23 (7.6)	22/276 (8.0)	1/25 (4.0)	p = 0.706
**Scrotal swelling**		***p = 0*.*012***			
No	10554 (98.5)	325 (3.1)	-	-	-
Yes	160 (1.5)	11 (6.9)	-	-	-
**Dysuria (Urinary pain)**		***p = 0*.*086***			
No	9509 (88.8)	309 (3.2)	240/7886 (3.0)	69/1623 (4.3)	**p = 0.017**
Yes	1205 (11.2)	51 (4.2)	48/1100 (4.4)	3/105 (2.9)	p = 0.610

*p-value statistics are from chi-square and Fisher’s exact tests; na = not applicable

**Other variables with p>0.20 for prevalence of HIV infection:** diabetes diagnosis (p = 0.62), cancer diagnosis (p = 0.50), prior prison duration (p = 57), current use of Khat (p = 0.36), residence prior to prison (p = 0.25), and number of people in the house (p = 0.41).

### Multivariable logistic regression model of risk factors for HIV infection by socio-demographic, incarceration, sexual behavior, and medical characteristics in all participants

In a multivariable logistic regression model (Table 4), multiple factors were significantly and independently associated with HIV infection as messured by adjusted odds ratios (AOR). These included: a) increasing number of lifetime sexual partners (p<0.001), b) age (AOR per age category ranging from 1.5–6.4, p<0.001), c) female sex (AOR = 2.5, p<0.001), d) marital status (p = 0.016) especially being divorced/separated (AOR = 2.7, p = 0.001), e) lifetime history of TB diagnosis (AOR = 5.9, p<0.001), f) syphilis (AOR = 4.6, p<0.001), and g) history of genital ulcer (AOR = 2.2, p = 0.037). Unexpectedly, dysuria, a common symptom of some sexually-transmitted infections, was associated with reduced risk of HIV infection (AOR = 0.49, p = 0.007) (Table 5).

**Table 4:** Prevalence of HIV in entrants and residents by screening period.

Screening wave / p values	E1 (n = 961)	E2 (n = 3,277)	EG (n = 1,782)	R1 (n = 2,651)	R2 (n = 2,043)	Total (N = 10,714)	E1 vs R1	E2 vs R2
% HIV Positives	21 (2.2%)	92 (2.8%)	48 (2.7%)	64 (2.4%)	111 (5.4%)	336 (3.1%)	*p = 0*.*80*	***p<0*.*001***

**E1/2 =** Entrants 1^st^/2^nd^ screening, **R1/2 =** Residents 1^st^/2^nd^ screening, **EG =** Entrants screened between E1 and E2

**Table 5:** Multivariable analysis of risk factors for all HIV infected participants.

Characteristics/Variable	*AOR	95% C.I.		p-value
		Lower	Upper	
**Gender**				
Male	1	-	-	**-**
Female	2.52	1.73	3.67	**<0.001**
**Age**				**<0.001**
18–20	1	-	-	-
21–25	1.49	0.87	2.56	0.148
26–30	2.39	1.39	4.11	**0.002**
31–35	3.46	1.92	6.26	**<0.001**
36–40	5.14	2.80	9.44	**<0.001**
41–45	6.40	3.28	12.5	**<0.001**
46 and above	3.30	1.72	6.31	**<0.001**
**Marital Status**				**0.016**
Never Married	1	-	-	-
Married	1.30	0.95	1.80	0.101
Divorced/Separated	2.71	1.49	4.93	**0.001**
Widow/Widower	2.45	0.78	7.73	0.126
Other	1.66	0.47	5.82	0.430
**TB diagnosis ever**				
No	1	-	-	-
Yes	5.92	4.03	8.70	**<0.001**
**Number of Sex Partners**				**<0.001**
Never	1	-	-	-
1 partner	0.85	0.50	1.47	0.565
2–10 partners	1.62	0.95	2.77	0.076
11 and above partners	1.21	0.59	2.50	0.610
Negative	1	-	-	**-**
Positive	4.59	3.18	6.64	**<0.001**
**Dysuria (Urinary pain)**				
No	1	-	-	**-**
Yes	0.49	0.29	0.83	**0.007**
**Genital ulcer**				
No	1	-	-	**-**
Yes	2.18	1.05	4.52	**0.037**

*AOR = Adjusted Odds Ratio; **95% C.I.** = 95% Confidence Interval

**Variables with p>0.05:** education (p = 0.33), occupation (p = 0.22), ethnicity (p = 0.50), religion (p = 0.14), BMI (p = 0.60), genital discharge (p = 0.10), scrotal swelling (p = 0.13), prior imprisonment (p = 0.85), prison location (p = 0.33), current prison duration (p = 0.16), current smoking (p = 0.43), and alcohol use prior to prison (p = 0.34).

### Gender differences in HIV prevalence by categories of socio-demographic, incarceration, sexual behavior, and medical characteristics

Comparisons in HIV prevalence rates by multiple categories identified many that differed between male and female prisoners (Table 3). Women were significantly more likely than men to be HIV+ in 4 of 6 age categories under 40 years, but not for the two categories >40 years (p = 0.001). Women were more likely to be HIV+ than men in ethnicity categories; Oromo and the “other.” HIV prevalence in women was higher than in men across all levels of education (p = 0.046), but rates were not significantly higher at any single educational level. The sexes did not differ overall in HIV prevalence by occupational categories. In the occupational category of “none”, women had the highest HIV prevalence and men the lowest (W 9.4 vs M 1.2%, p = 0.003. an 8-fold difference. However, numbers of HIV+ prisoners in this “none” category were small (3 men and 6 women).

No marital status category had a statistical significance diference in HIV prevalence by sex. However, a greater proportion of women who were unmarried (W 3.0 vs M 2.0%) or who were divorced / separated (W 20 vs M 11.5%) were HIV+ compared to their male counterparts. Women residing in urban settings before imprisonment were more likely to be HIV+ than urban men (4.3 vs 3.1, p = 0.013). Women living prior to imprisonment in either a one-room residence (6.0 vs 3.3%, p = 0.001) or a 6–10 room residence (6.1 vs 2.8%, p = 0.038) were more likely to be HIV+ than equivalently-housed men.

Women entering prison were more likely to be HIV+ than entering men (W 5.5% vs M 2.5%, p< 0.001). For residents the difference was reversed, but not significant so (3.2%in women vs 4.3% in men, p = 0.125). Previously imprisoned women tended to have higher HIV+ prevalence than similar men, but this difference was of only borderline significance (4.1 vs 3.1, p = 0.059). Most Kaliti prisoners had been imprisoned for <5 years (92.6%) and in this group women were significantly more likely to be infected (4.2 vs 3.1%, p = 0.021). The number of lifetime sexual partners was a greater risk factor for HIV infection in women than men for the largest two categories: 2–10 (9.4 vs 4.2%, p<0.001) and >10 (20.0 vs 4.5%, p = 0.033). For symptoms of current genital infections (discharge, ulcers, or dysuria), HIV infection was more common in women, but not in men, with these symptoms.

## Discussion

### Prevalence of HIV infection in all prisoners and in entrants and residents

As expected, this study found that the overall HIV prevalence in a large central prison (3.4%) was higher than in the general population of Ethiopia (2.0%). HIV prevalence varies markedly among Ethiopian and other African prisons. For example, HIV prevalence at Kality (3.4%) was 12-fold lower than that in Swaziland correctional services (40%) [13] and 1.7-fold lower than that in Ghanaian prisons (5.9%) [14]. It was also lower than in two regional prisons in Ethiopia from a northern region (Woldiya = (6.3%) (15) and southern region (Tabor/Hawassa) prison = 6.0%) [15]. In contrast, prevalence of HIV in a south central prison (Jimma Regional) was lower than Kaliti’s (2.6%) [16]. These differences within Ethiopia may reflect the differing prevalences of HIV in the general populations of various regions of Ethiopia: for example, 4.1% in Amhara (north-central), 1.8% in the SNNPR (south), and 3.0% in Oromia (south-central) [17].

We hypothesized that HIV prevalence rates in the first screening wave would be higher in residents (R1) than entrants (E1) because of intramural HIV transmission. However, differences in HIV prevalence at our first screening (i.e., between E1 and R1) were small and not statistically significant. In contrast, in the second wave, resident prisoners (R2) had significantly higher rates than entrants (E2). Thus, findings only in the second wave were consistence with our hypothesis that intramural transmission of STIs would result in a higher prevelance in residents than entrants.

The reason for this unexpected increase in HIV prevalence in residents between the two screening periods is unclear. A possible explanation is that a subgroup of inmates with either known HIV infection or high risk of HIV did not participate in the R1. They may have feared that a diagnosis of HIV would lead to adverse consequences such as shunning by other prisoners or mistreatment by prison authorities. Some of them may have volunteered for R2 after being reassured by the lack of such consequences for prisoners participating in R1. Less likely explanations are a substantial increase in sexual activity within the prison between waves or errors in laboratory testing. These seem unlikely because conditions in the prison and its laboratory did not change substantially during this period.

Our second hypothesis was that HIV prevalence would decline in residents between the two waves of screening because treatment of HIV-infected residents and entrants would reduce transmission within the prison by reducing HIV in their bood and genital fluids. However, this hypothesis was not supported by our findings. In fact, HIV prevalence was greater in R2 than in R1 by 2.3-fold. Inaccuracy in prevalence estimates in residents for reasons discussed above may have obscured any beneficial effects of screening.

### Risk factors for HIV infection

Risk factors that were independently associated with HIV infection in all prisoners in a multivariable analysis were a) increasing age, b) female gender, c) smaller number of rooms in their pre-prison residence, d) marital status (being a widow/widower, divorced or separated, p = 0.016), e) TB diagnosis ever, f) number of lifetime sex partners (especially having 2–10), g) history of genital ulcer(s), and h) reaginic antibody evidence of latent syphilis. Many of these factors reflect either lower socio-economic status or greater exposure to sexually transmitted diseases.

Risk factors for HIV in Kality prisoners were similar to those in the general Ethiopian population [18]. HIV prevalence was 1.3-fold higher in prisoners who resided in urban than rural areas. This difference is less than the 7-fold greater prevalence of HIV in urban than rural dwellers in the general Ethiopian population [19]. Large differences in HIV prevalence were found in several categories of marital status: divorced/separated (13.1% vs 2.9%), married (4.3% vs 0.8%) and never married (2.1% vs 1.0%), except widowers (10.2% vs 11.5%). Prevalence of HIV increased with the number of lifetime sex partners and the current duration of imprisonment. Neither prior imprisonment nor its duration influenced the prevalence of HIV positivity.

Housewives, divorced / separated persons or widows/widowers and the better educated (graduates of preparatory school or more) had higher HIV prevalence than other occupations, marital groups, and educational categories. Consistent with this pattern, prevalence of HIV was highest at the extremes of education and occupational categories i.e. higher in the more educated (merchants and government employees) and the least educated (peasants and those with no formal education) than those with intermediate levels of education. The reasons for this non-linear (U-shaped) distribution are unclear, but may be related to greater access to multiple sexual partners by the more affluent and by the poor than by other Ethiopians, but no direct evidence supports this hypotheses. We found that a history of symptoms of sexually transmitted infections such as scrotal swelling, genital discharge, and genital ulcer or of syphilis were associated with HIV infection in men, but not in women.

Risk factors for HIV infections in Ethiopian prisoners in Jimma Regional Prison were similar to those that we found [19]. They included: a) male sex, b) age > 50 years, c) primary or less school education, d) history of a blood transfusion (a factor we did not address), and e) serologic diagnosis of syphilis

### Gender differences in HIV prevalence by categories of socio-demographic, incarceration, sexual behavior, and medical characteristics

Prevalence of HIV in the female Kaliti prisoners in this study (72/1728 = 4.2%) was higher than that for men (3.2%).(Table 2) A review of HIV epidemiology in Addis Ababa emphasized the role of female sex workers and “hot spots in transmission [20]. Thus, female prisoners might be at higher risk because they were more likely to have been sex workers than other Ethiopian woman. However, we could not examine that possibility because we were not permitted to collect data on that risk factor. Unexpectedly, HIV prevalence in the imprisoned women was slightly lower than that estimated for contemporaneously studied (2014), mainly urban, pregnant, Ethiopian women (5.5%) [3]. Thus, they appear no more likely to have been HIV infected than other women.

Many characteristics differed between male and female prisoners. The prevalence of HIV was strikingly 2.2-fold higher in women than men entering Kality Prison (W = 5.5% > M = 2.5%, p<0.001). The ratio was reversed in residents (W = 3.2% < M = 4.3,% p = 0.125). Reasons for this large difference between entrants and residents in the ratio of HIV prevalence in men and women is unclear, but include differences between the sexes in participation by HIV+ prisoners in the study or their management such as release or transfer to other prisons.

HIV+ was more prevalent in female than male prisoners in multiple demographic, behavioral, and serological categories consistent with increased exposure to HIV and other sexually transmitted diseases. Risk factors indicating a higher risk for HIV infection in women than in men included: a) age < 40 years, b) endorsed Oromo and the “other” categories of Ethiopian ethnicities, c) urban origins, d) unmarried divorced or separated, e) had been previously imprisoned, and f) had more than two lifetime sexual partners. They were more likely than men to be HIV+ across all levels of education, but not by occupational categories.

A studies 1679 workers in two Ethiopian factories earlier period of the HIV epidemic (1997) found very higher HIV prevalence in both men (~ 10%) and women (~12%) [21]. Risk factors for HIV were similar to those in Kaliti prisoners 17 years later. In this earlier study the major self-reported risk factors were indicators of high-risk sexual behaviors such as a) higher lifetime sexual partners in men, b) low income history of rape and casual sex in women and c) serologic evidence of sexually transmitted diseases (syphilis and herpes simplex virus, type 2) in both sexes.

The prevalence of HIV was significantly higher in this prison than in the general population confirming that prisoners are a high-risk group for HIV in Ethiopia as they are globally. The higher prevalence of HIV in prisoners than the general population confirms that HIV screening and treatment of prisoners should be a priority. Prevalence rates were higher in resident than entrant prisoners in only one of two periods that we surveyed not consistently supporting or falsifying our hypothesis that intramural transmission of HIV was occurring. Moreover, HIV prevalence increased in residents after screening and treating both entrants and residents for over a year. This finding does not support our second hypotheses that screening for and treating HIV infection would reduce its prevalence in this prison.

HIV prevalence was higher in female than male prison entrants, consistent with the pattern in the general population requiring that they be similarly screened and treated of HIV infection. HIV prevalence in residents of both sexes were similar, an unexplained difference in pattern from that in entrants.

### Strengths and limitations

The strengths of this study included: a) inclusion of large numbers of both residents and entrants with wide demographic diversity and b) two cross-sectional waves of screenings of residents and continuous screening of entrants. Our study had the following limitations.

First, participation was voluntary and some entrants were not even approached because they arrived and were processed on weekends or at times the study staff were not available. Second, no unique identifying number was available to us that would allow us to link data on prisoners seen multiple times in the study. Moreover, prisoners were frequently transferred to other prisons or released between the two cross-sectional waves of screening. Thus, we were unable to assess prisoners longitudinally and settled for two cross-sectional waves of assessment of residents.

Third, we did not collect data on blood-related activities associated with transmission (e.g. tattooing or transfusions) and were not allowed to ask about sexual risk activities during imprisonment. Fourth, for security reasons study investigators and research staff who were not prison employees were denied access to high-security areas of the prison where the residents were screened. Thus, they could not directly evaluate the quality of recruitment, interviewing, or assessment methods employed in residents by the prison medical staff. However, we were allowed to observe and assess the screening of entrants. Fifth, we had no access to data on the daily numbers or rates of new entrants, census of residents, or transfers to or from the prison.

## Conclusions

The prevalence of HIV was significantly higher in this prison than in the general population confirming that prisoners are a high-risk group for HIV in Ethiopia as they are globally. The higher prevalence of HIV in prisoners than the general population confirms that HIV screening and treatment of prisoners should 452 be a priority. Prevalence rates were higher in resident than entrant prisoners in only the second of two periods that we surveyed. Thus, our hypothesis that intramural transmission of HIV would increase prevalence in residents compared with entrants was neither consistently supported or falsified. Moreover, HIV prevalence increased in residents after screening and treating both entrants and residents for over a year. This finding does not support our second hypotheses that screening for and treating HIV infection would reduce its prevalence in this prison. HIV prevalence was higher in women than men entering prison, consistent with the pattern in the general population. The prevalence in entering women was similar to that of pregnant women in the general population attending antennal clinics, supporting they were not a greater risk of HIV before imprisonment, but HIV prevalence in residents of both sexes were similar suggesting that they were a risk during imprisonment. Multiple limitations the design and implementation of the study listed above contributed to problems that may have compromised the accuracy of our estimates of HIV prevalence and their implications.

## Supporting information

S1 Data (XLSX)

S2 Data (XLSX)

## References

[1] UNAIDS. Global HIV & AIDS statistics: 2021 fact sheet. Geneva: https://embargo.unaids.org/static/files/uploaded_files/UNAIDS_2021_Fact

[2] World Prison Populations List, 2021 Institute for Crime & Justice Policy Research (ICPR) at Birkbeck, University of London https://www.prisonstudies.org/sites/default/files/resources/downloads/world_prison_population_list_13th_edition.pdf

[3] Kassa D, Gebremichael G, Tilahun T, Ayalkebet A, Abrha Y, et al Prevalence of sexually transmitted infections (HIV, hepatitis B virus, herpes simplex virus type 2, and syphilis) in pregnant women in Ethiopia: Trends over 10 years (2005–2014) 2019 Int *J Infect Dis* 79:50–57 10.1016/j.ijid.2018.11.009 30472433

[4] Golrokhi Raheleh, Farhoudi Behnam, Taj Leila, Fatemeh Golsoorat Pahlaviani, et al HIV Prevalence and Correlations in Prisons in Different Regions of the World: A Review Article 2018 *The Open AIDS Journal* 12:81–92 doi: 10.2174/1874613601812010081 30369993 PMC6176549

[5] Report on the 2014 Round Antenatal Care based Sentinel HIV Surveillance in Ethiopia. *The Ethiopian Public Health Institute*, Addis Ababa July 2015.

[6] Kebede Y, Pickering J, McDonald J, Zewde D, et al HIV Infection in an Ethiopian Prison. *Am*. *J*. *Public Health* 1991; 81:625–627. doi: 10.2105/ajph.81.5.625 2014865 PMC1405083

[7] Sahle ET, Blumenthal J, Jain S, Sun S, Young J, Amogne W, et al. Bacteriologically-confirmed pulmonary tuberculosis in an Ethiopian prison: Prevalence from screening of entrant and resident prisoners 2019 *PLoS One*; 14: e0226160. doi: 10.1371/journal.pone.0226160 31830092 PMC6907752

[8] MOH (2007). Guidelines for HIV counseling and testing in Ethiopia. Addis Ababa: MOH. http://www.who.int/hiv/topics/vct/ETH_HCT_guidelinesJune26_clean.pdf

[9] KHB (HIV (1+2) Antibody Colloidal Gold) rapid test. Shanghai Kehua Bioengineering, Ltd, Shanghai, China. Available at: https://www.who.int/diagnostics_laboratory/evaluations/pq-list/hiv-rdts/161221_final_public_report_0267_037_00.pdf?ua=1.

[10] HIV 1/2 STAT-PAK®. Chembio Diagnostic Systems, Inc. Medford, NY, USA. Available at: http://chembio.com/wp-content/uploads/2018/10/10-6072-1-Product-Insert-for-HIV-12-STAT-PAK-Assay-FG-60-9505-1-Rev-2.pdf.

[11] World Health Organization (WHO)/Joint United Nations Programme on HIV/AIDS (UNAIDS). 2002. HIV Simple/Rapid Assays: Operational Characteristics (Phase 1): Report 12, Whole Blood Specimens. *WHO/BCT/02*.*07*. Geneva: WHO.

[12] Guidelines for implementation of the antiretroviral therapy program in Ethiopia from the Federal HIV/AIDS Prevention and Control Office Federal Ministry of Health, July 2007

[13] Happiness Mkhatshwa. The Assessment of HIV/Syphilis/PTB/Hepatitis Prevalence in His Majesty’s Correctional Services Setting in Swaziland, 2010. February 2011 (Unpublished manuscript).

[14] Adjei Andrew A, Armah Henry B, Gbagbo Foster, Ampofo William K, Boamah Isaac, Adu-Gyamfi Clement, et al. Correlates of HIV, HBV, HCV and syphilis infections among prison inmates and officers in Ghana: A national multicenter study. BMC Infectious Diseases 2008, 8:33 doi: 10.1186/1471-2334-8-33 PMC2311310 18328097

[15] Moges B, Amare B, Asfaw F, Tesfaye W, Tiruneh M, Belayhun Y, et al. Prevalence of smear positive pulmonary tuberculosis among prisoners in North Gondar zone prison, northwest Ethiopia. *BMC Infectious Diseases*. 2012;12:352 doi: 10.1186/1471-2334-12-352 23241368 PMC3531261

[16] Busi S, Oltaye Z. Assessment of magnitude of sexually transmitted infections, sexual and reproductive health status among prisoners aged between 18–49 years in Tabor Prison, Hawassa, Ethiopia. *Ethiopian J Sci* 2016; 8(1): 89–97.

[17] Ethiopian Population-based HIV Impact Assessment (EPHIA). Summary Sheet: Preliminary Findings. EPHIA 2017–2018. https://phia.icap.columbia.edu/wp-content/uploads/2018/12/3511%E2%80%A2EPHIA-Summary-Sheet_v30.pdf.

[18] Hailu BA, Tadese F, Bogale GG, Molla A, Miheretu BA, and Beyene J Spatial patterns and associated factors of HIV Seropositivity among adults in Ethiopia from EDHS 2016: a spatial and multilevel analysis *BMC Infect Dis*. 2020; 20: 751. doi: 10.1186/s12879-020-05456-y 33054788 PMC7557038

[19] Kebede W, Abdissa A, Seid Y, Mekonnen Z. Seroprevalence and risk factors of hepatitis B, hepatitis C and HIV infections among prisoners in Jimma Town, Southwest Ethiopia. *Asian Pacific Journal of Tropical Disease* 2017; doi: 10.12980/apjtd.7.2017D6-422

[20] Kibret GD, Ferede A, Leshargie CT, Wagnew F, Ketema DB, and Alebel A Trends and spatial distributions of HIV prevalence in Ethiopia *Infect Dis Poverty*. 2019; 8: 90 doi: 10.1186/s40249-019-0594-9 31623689 PMC6796490

[21] Mekonnen Y, Sanders E, Messele T, Wolday D, Dorigo-Zestma W, et al Prevalence and incidence of, and risk factors for, HIV-1 infection among factory workers in Ethiopia, 1997–2001 *J Health Popul Nut* 2005 23:358–68. 16599107

